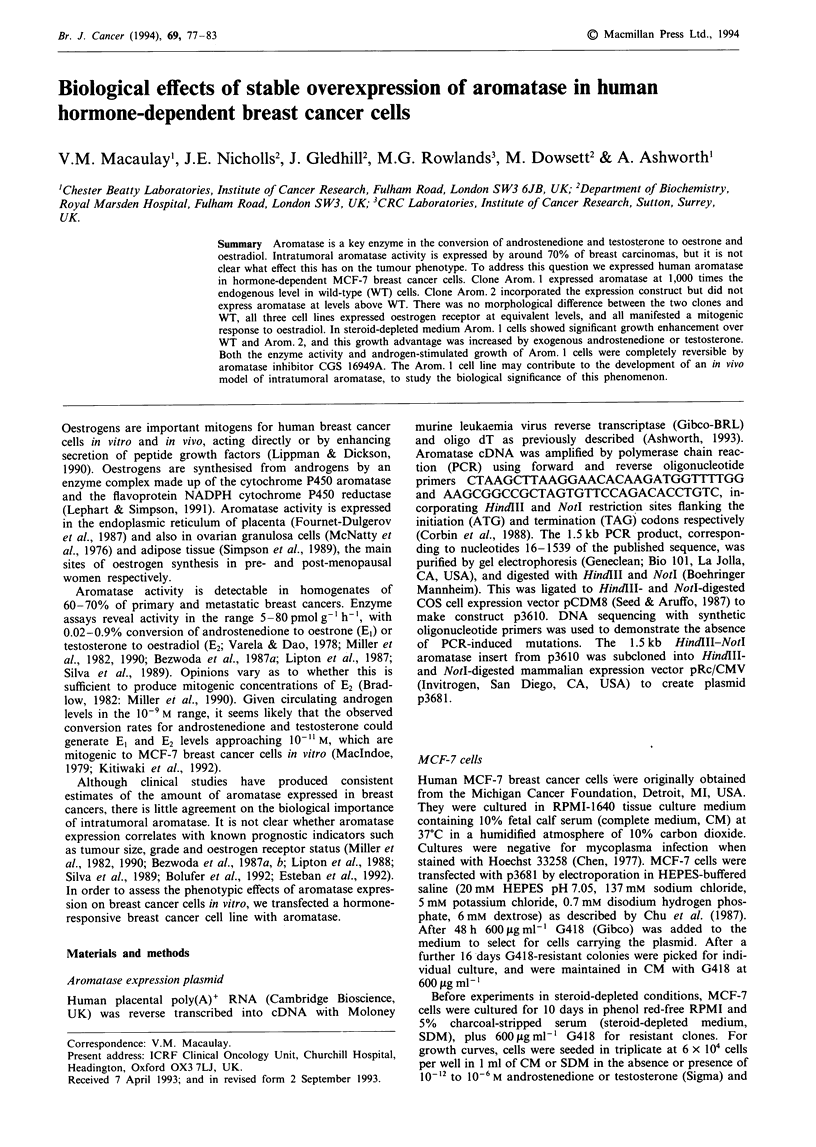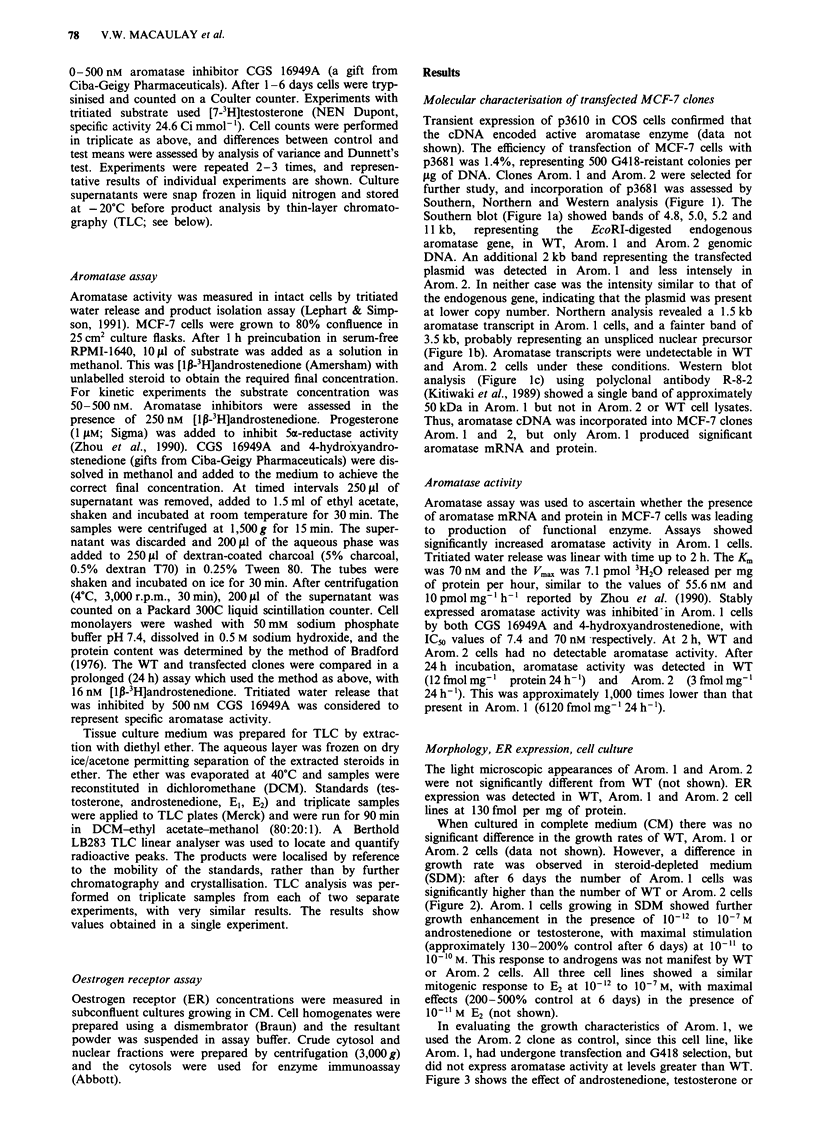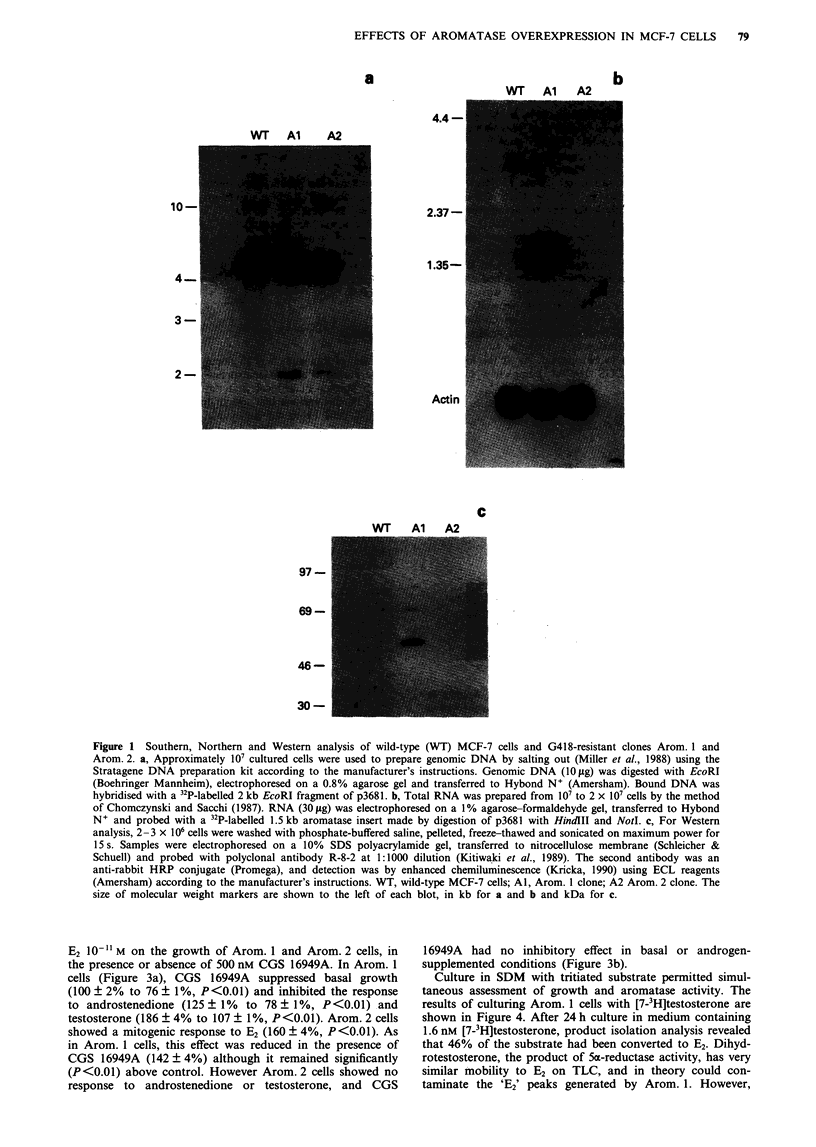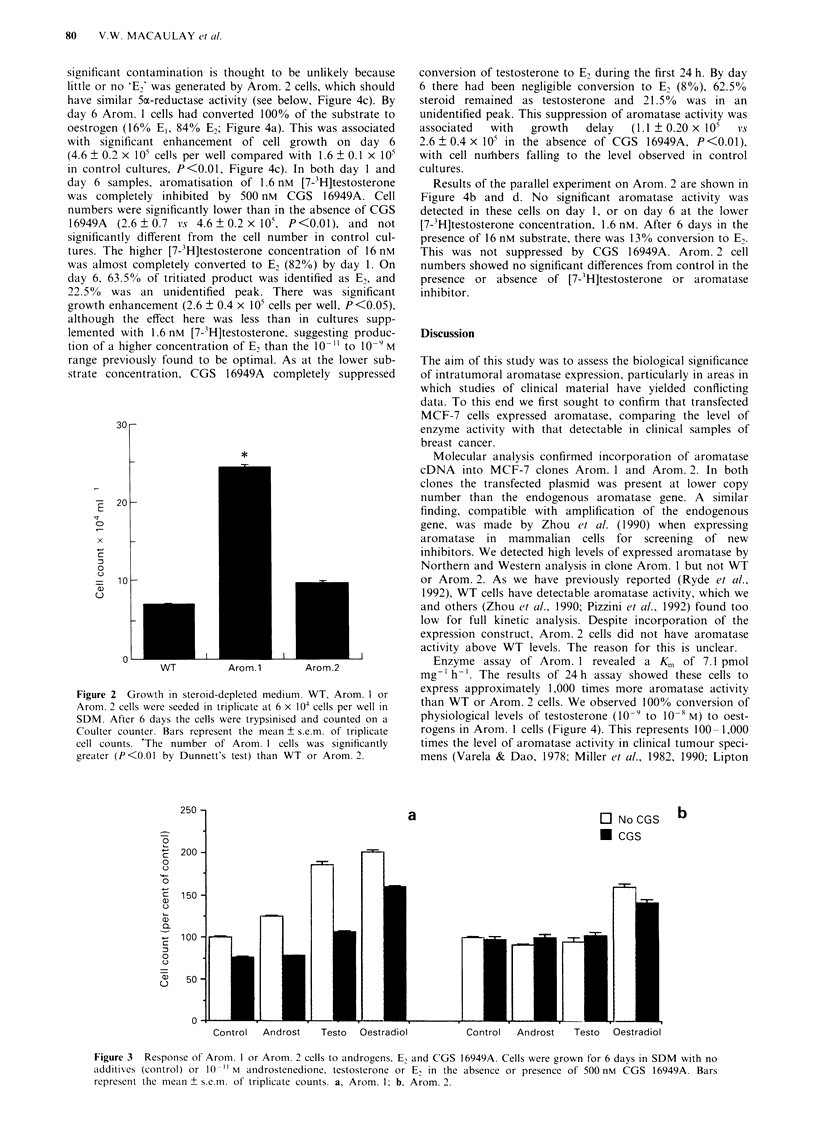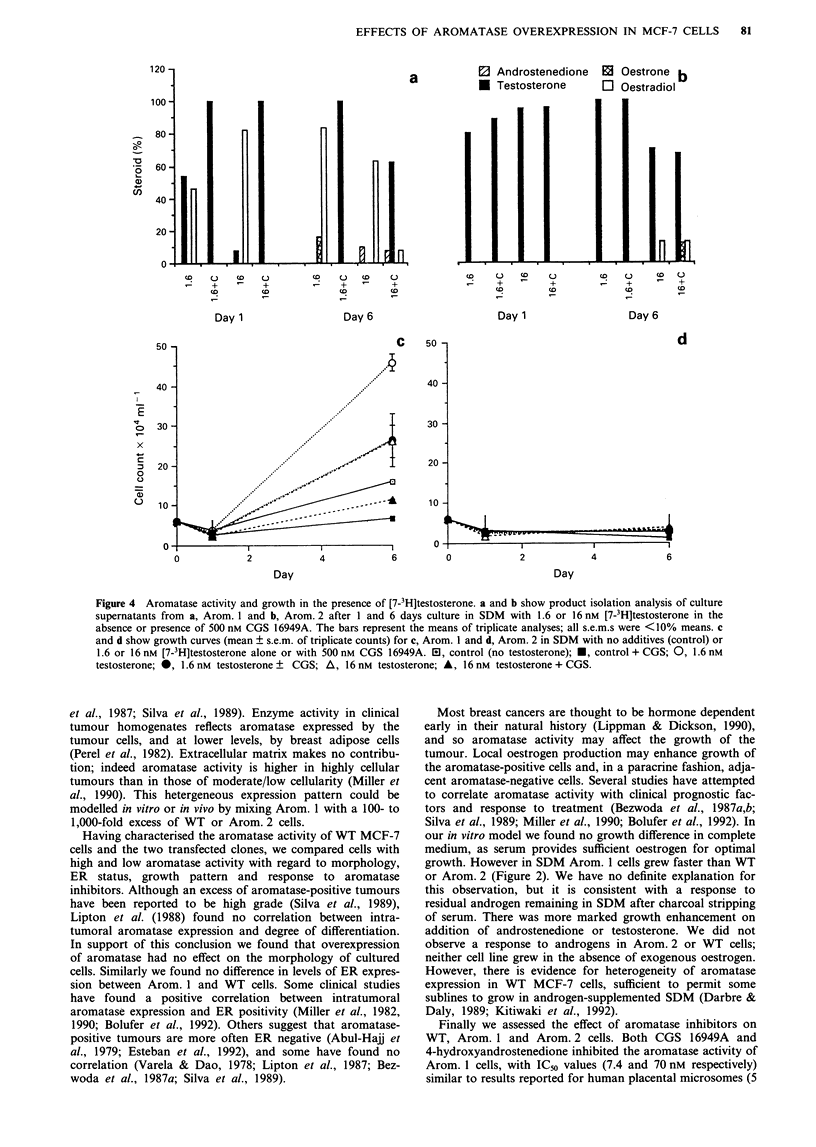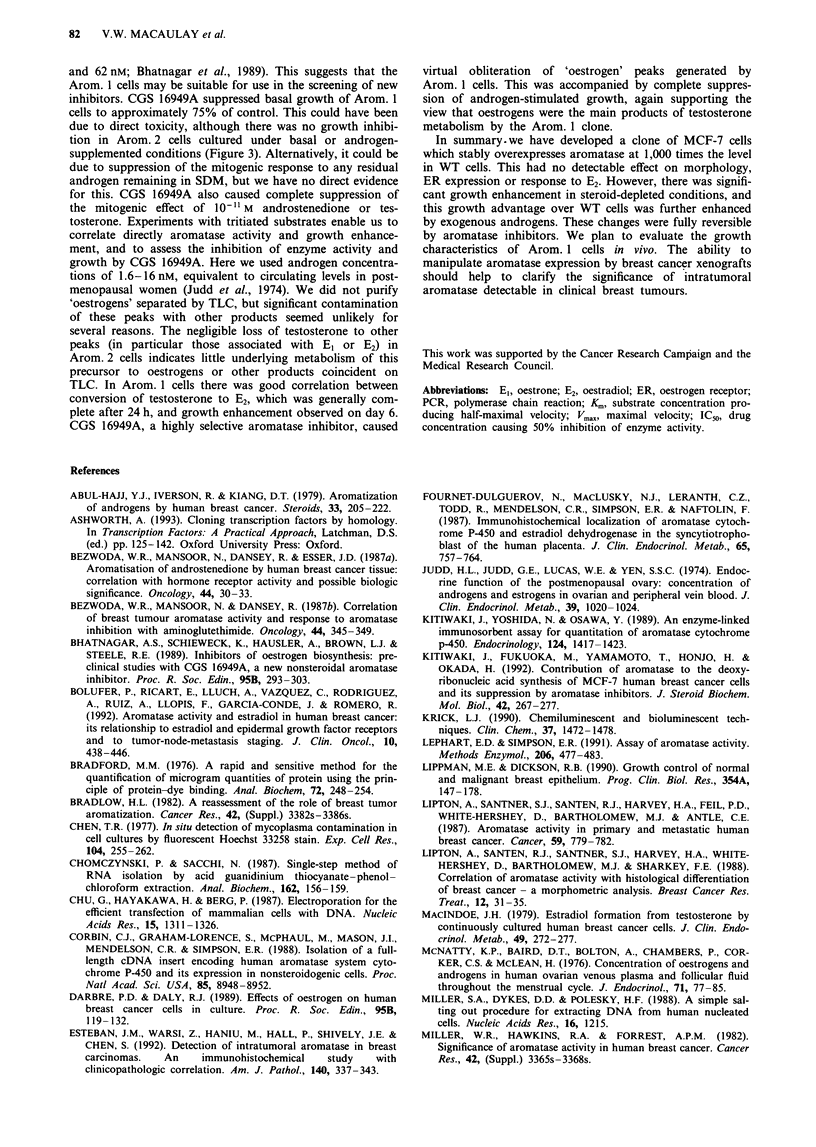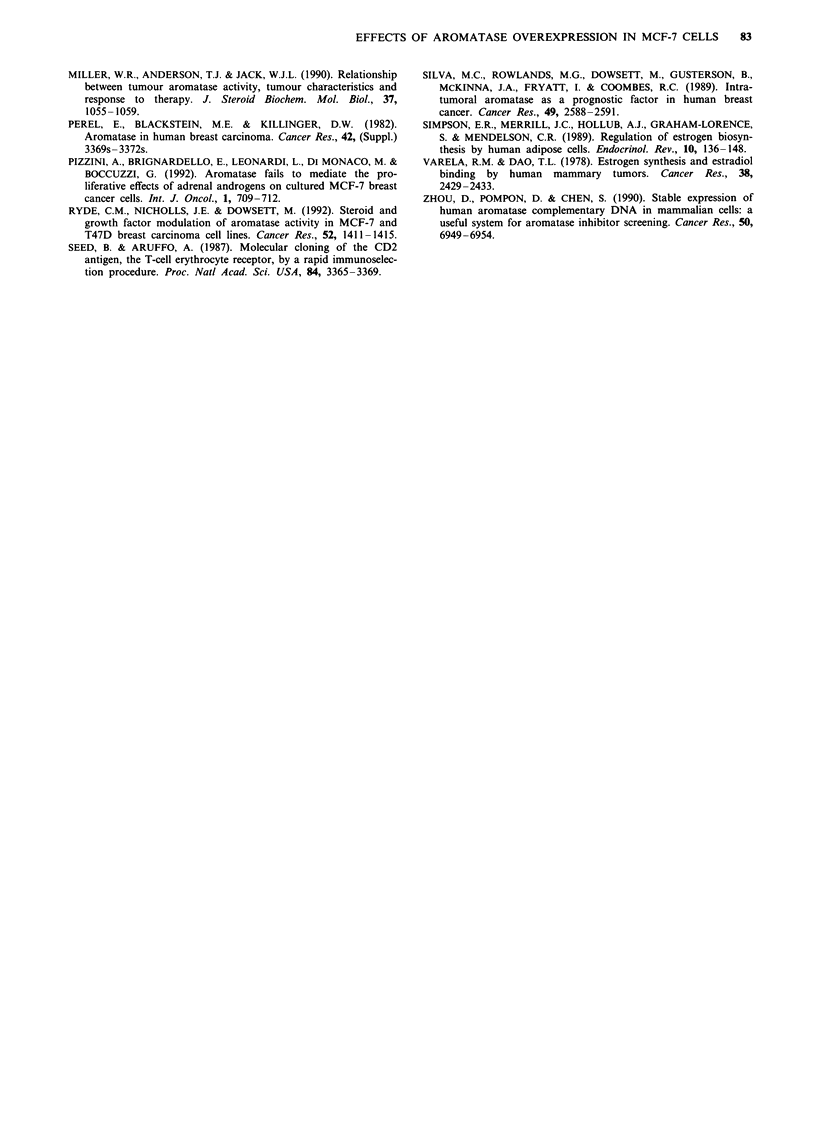# Biological effects of stable overexpression of aromatase in human hormone-dependent breast cancer cells.

**DOI:** 10.1038/bjc.1994.12

**Published:** 1994-01

**Authors:** V. M. Macaulay, J. E. Nicholls, J. Gledhill, M. G. Rowlands, M. Dowsett, A. Ashworth

**Affiliations:** Chester Beatty Laboratories, Institute of Cancer Research, London, UK.

## Abstract

**Images:**


Br. J. Cancer (1994), 69, 77-83 © Macmillan Press Ltd., 1994

### Biological effects of stable overexpression of aromatase in human hormone-dependent breast cancer cells

V.M. Macaulay[1], J.E. Nicholls[2], J. Gledhill[2], M.G. Rowlands[3], M. Dowsett[2] & A. Ashworth[1]

[1]*Chester Beatty Laboratories, Institute of Cancer Research, Fulham Road, London SW3 6JB, UK;* [2]*Department of Biochemistry, Royal Marsden Hospital, Fulham Road, London SW3, UK;* [3]*CRC Laboratories, Institute of Cancer Research, Sutton, Surrey, UK.*

**Summary** Aromatase is a key enzyme in the conversion of androstenedione and testosterone to oestrone and oestradiol. Intratumoral aromatase activity is expressed by around 70% of breast carcinomas, but it is not clear what effect this has on the tumour phenotype. To address this question we expressed human aromatase in hormone-dependent MCF-7 breast cancer cells. Clone Arom. 1 expressed aromatase at 1,000 times the endogenous level in wild-type (WT) cells. Clone Arom. 2 incorporated the expression construct but did not express aromatase at levels above WT. There was no morphological difference between the two clones and WT, all three cell lines expressed oestrogen receptor at equivalent levels, and all manifested a mitogenic response to oestradiol. In steroid-depleted medium Arom. 1 cells showed significant growth enhancement over WT and Arom. 2, and this growth advantage was increased by exogenous androstenedione or testosterone. Both the enzyme activity and androgen-stimulated growth of Arom. 1 cells were completely reversible by aromatase inhibitor CGS 16949A. The Arom. 1 cell line may contribute to the development of an *in vivo* model of intratumoral aromatase, to study the biological significance of this phenomenon.

Oestrogens are important mitogens for human breast cancer cells *in vitro* and *in vivo*, acting directly or by enhancing secretion of peptide growth factors (Lippman & Dickson, 1990). Oestrogens are synthesised from androgens by an enzyme complex made up of the cytochrome P450 aromatase and the flavoprotein NADPH cytochrome P450 reductase (Lephart & Simpson, 1991). Aromatase activity is expressed in the endoplasmic reticulum of placenta (Fournet-Dulgerov *et al.*, 1987) and also in ovarian granulosa cells (McNatty *et al.*, 1976) and adipose tissue (Simpson *et al.*, 1989), the main sites of oestrogen synthesis in pre- and post-menopausal women respectively.

Aromatase activity is detectable in homogenates of 60-70% of primary and metastatic breast cancers. Enzyme assays reveal activity in the range 5-80 pmol $g^{-1}$ $h^{-1}$, with 0.02-0.9% conversion of androstenedione to oestrone ($E_1$) or testosterone to oestradiol ($E_2$; Varela & Dao, 1978; Miller *et al.*, 1982, 1990; Bezwoda *et al.*, 1987*a*; Lipton *et al.*, 1987; Silva *et al.*, 1989). Opinions vary as to whether this is sufficient to produce mitogenic concentrations of $E_2$ (Bradlow, 1982: Miller *et al.*, 1990). Given circulating androgen levels in the $10^{-9}$ M range, it seems likely that the observed conversion rates for androstenedione and testosterone could generate $E_1$ and $E_2$ levels approaching $10^{-11}$ M, which are mitogenic to MCF-7 breast cancer cells *in vitro* (MacIndoe, 1979; Kitiwaki *et al.*, 1992).

Although clinical studies have produced consistent estimates of the amount of aromatase expressed in breast cancers, there is little agreement on the biological importance of intratumoral aromatase. It is not clear whether aromatase expression correlates with known prognostic indicators such as tumour size, grade and oestrogen receptor status (Miller *et al.*, 1982, 1990; Bezwoda *et al.*, 1987*a*, *b*; Lipton *et al.*, 1988; Silva *et al.*, 1989; Bolufer *et al.*, 1992; Esteban *et al.*, 1992). In order to assess the phenotypic effects of aromatase expression on breast cancer cells *in vitro*, we transfected a hormone-responsive breast cancer cell line with aromatase.

### Materials and methods

### *Aromatase expression plasmid*

Human placental poly(A)$^+$ RNA (Cambridge Bioscience, UK) was reverse transcribed into cDNA with Moloney murine leukaemia virus reverse transcriptase (Gibco-BRL) and oligo dT as previously described (Ashworth, 1993). Aromatase cDNA was amplified by polymerase chain reaction (PCR) using forward and reverse oligonucleotide primers CTAAGCTTAAGGAACACAAGATGGTTTTGG and AAGCGGCCGCTAGTGTTCCAGACACCTGTC, incorporating *Hind*III and *Not*I restriction sites flanking the initiation (ATG) and termination (TAG) codons respectively (Corbin *et al.*, 1988). The 1.5 kb PCR product, corresponding to nucleotides 16-1539 of the published sequence, was purified by gel electrophoresis (Geneclean; Bio 101, La Jolla, CA, USA), and digested with *Hind*III and *Not*I (Boehringer Mannheim). This was ligated to *Hind*III- and *Not*I-digested COS cell expression vector pCDM8 (Seed & Aruffo, 1987) to make construct p3610. DNA sequencing with synthetic oligonucleotide primers was used to demonstrate the absence of PCR-induced mutations. The 1.5 kb *Hind*III-*Not*I aromatase insert from p3610 was subcloned into *Hind*III- and *Not*I-digested mammalian expression vector pRc/CMV (Invitrogen, San Diego, CA, USA) to create plasmid p3681.

### *MCF-7 cells*

Human MCF-7 breast cancer cells were originally obtained from the Michigan Cancer Foundation, Detroit, MI, USA. They were cultured in RPMI-1640 tissue culture medium containing 10% fetal calf serum (complete medium, CM) at 37°C in a humidified atmosphere of 10% carbon dioxide. Cultures were negative for mycoplasma infection when stained with Hoechst 33258 (Chen, 1977). MCF-7 cells were transfected with p3681 by electroporation in HEPES-buffered saline (20 mM HEPES pH 7.05, 137 mM sodium chloride, 5 mM potassium chloride, 0.7 mM disodium hydrogen phosphate, 6 mM dextrose) as described by Chu *et al.* (1987). After 48 h 600 $\mu g$ $ml^{-1}$ G418 (Gibco) was added to the medium to select for cells carrying the plasmid. After a further 16 days G418-resistant colonies were picked for individual culture, and were maintained in CM with G418 at 600 $\mu g$ $ml^{-1}$

Before experiments in steroid-depleted conditions, MCF-7 cells were cultured for 10 days in phenol red-free RPMI and 5% charcoal-stripped serum (steroid-depleted medium, SDM), plus 600 $\mu g$ $ml^{-1}$ G418 for resistant clones. For growth curves, cells were seeded in triplicate at $6 \times 10^4$ cells per well in 1 ml of CM or SDM in the absence or presence of $10^{-12}$ to $10^{-6}$ M androstenedione or testosterone (Sigma) and

Correspondence: V.M. Macaulay.
Present address: ICRF Clinical Oncology Unit, Churchill Hospital, Headington, Oxford OX3 7LJ, UK.
Received 7 April 1993; and in revised form 2 September 1993.

0–500 nM aromatase inhibitor CGS 16949A (a gift from Ciba-Geigy Pharmaceuticals). After 1–6 days cells were trypsinised and counted on a Coulter counter. Experiments with tritiated substrate used [7-$^3$H]testosterone (NEN Dupont, specific activity 24.6 Ci mmol$^{-1}$). Cell counts were performed in triplicate as above, and differences between control and test means were assessed by analysis of variance and Dunnett's test. Experiments were repeated 2–3 times, and representative results of individual experiments are shown. Culture supernatants were snap frozen in liquid nitrogen and stored at −20°C before product analysis by thin-layer chromatography (TLC; see below).

*Aromatase assay*

Aromatase activity was measured in intact cells by tritiated water release and product isolation assay (Lephart & Simpson, 1991). MCF-7 cells were grown to 80% confluence in 25 cm$^2$ culture flasks. After 1 h preincubation in serum-free RPMI-1640, 10 µl of substrate was added as a solution in methanol. This was [1β-$^3$H]androstenedione (Amersham) with unlabelled steroid to obtain the required final concentration. For kinetic experiments the substrate concentration was 50–500 nM. Aromatase inhibitors were assessed in the presence of 250 nM [1β-$^3$H]androstenedione. Progesterone (1 µM; Sigma) was added to inhibit 5α-reductase activity (Zhou *et al*., 1990). CGS 16949A and 4-hydroxyandrostenedione (gifts from Ciba-Geigy Pharmaceuticals) were dissolved in methanol and added to the medium to achieve the correct final concentration. At timed intervals 250 µl of supernatant was removed, added to 1.5 ml of ethyl acetate, shaken and incubated at room temperature for 30 min. The samples were centrifuged at 1,500 *g* for 15 min. The supernatant was discarded and 200 µl of the aqueous phase was added to 250 µl of dextran-coated charcoal (5% charcoal, 0.5% dextran T70) in 0.25% Tween 80. The tubes were shaken and incubated on ice for 30 min. After centrifugation (4°C, 3,000 r.p.m., 30 min), 200 µl of the supernatant was counted on a Packard 300C liquid scintillation counter. Cell monolayers were washed with 50 mM sodium phosphate buffer pH 7.4, dissolved in 0.5 M sodium hydroxide, and the protein content was determined by the method of Bradford (1976). The WT and transfected clones were compared in a prolonged (24 h) assay which used the method as above, with 16 nM [1β-$^3$H]androstenedione. Tritiated water release that was inhibited by 500 nM CGS 16949A was considered to represent specific aromatase activity.

Tissue culture medium was prepared for TLC by extraction with diethyl ether. The aqueous layer was frozen on dry ice/acetone permitting separation of the extracted steroids in ether. The ether was evaporated at 40°C and samples were reconstituted in dichloromethane (DCM). Standards (testosterone, androstenedione, $E_1$, $E_2$) and triplicate samples were applied to TLC plates (Merck) and were run for 90 min in DCM–ethyl acetate–methanol (80:20:1). A Berthold LB283 TLC linear analyser was used to locate and quantify radioactive peaks. The products were localised by reference to the mobility of the standards, rather than by further chromatography and crystallisation. TLC analysis was performed on triplicate samples from each of two separate experiments, with very similar results. The results show values obtained in a single experiment.

*Oestrogen receptor assay*

Oestrogen receptor (ER) concentrations were measured in subconfluent cultures growing in CM. Cell homogenates were prepared using a dismembrator (Braun) and the resultant powder was suspended in assay buffer. Crude cytosol and nuclear fractions were prepared by centrifugation (3,000 *g*) and the cytosols were used for enzyme immunoassay (Abbott).

### Results

*Molecular characterisation of transfected MCF-7 clones*

Transient expression of p3610 in COS cells confirmed that the cDNA encoded active aromatase enzyme (data not shown). The efficiency of transfection of MCF-7 cells with p3681 was 1.4%, representing 500 G418-reistant colonies per µg of DNA. Clones Arom. 1 and Arom. 2 were selected for further study, and incorporation of p3681 was assessed by Southern, Northern and Western analysis (Figure 1). The Southern blot (Figure 1a) showed bands of 4.8, 5.0, 5.2 and 11 kb, representing the *Eco*RI-digested endogenous aromatase gene, in WT, Arom. 1 and Arom. 2 genomic DNA. An additional 2 kb band representing the transfected plasmid was detected in Arom. 1 and less intensely in Arom. 2. In neither case was the intensity similar to that of the endogenous gene, indicating that the plasmid was present at lower copy number. Northern analysis revealed a 1.5 kb aromatase transcript in Arom. 1 cells, and a fainter band of 3.5 kb, probably representing an unspliced nuclear precursor (Figure 1b). Aromatase transcripts were undetectable in WT and Arom. 2 cells under these conditions. Western blot analysis (Figure 1c) using polyclonal antibody R-8-2 (Kitiwaki *et al*., 1989) showed a single band of approximately 50 kDa in Arom. 1 but not in Arom. 2 or WT cell lysates. Thus, aromatase cDNA was incorporated into MCF-7 clones Arom. 1 and 2, but only Arom. 1 produced significant aromatase mRNA and protein.

*Aromatase activity*

Aromatase assay was used to ascertain whether the presence of aromatase mRNA and protein in MCF-7 cells was leading to production of functional enzyme. Assays showed significantly increased aromatase activity in Arom. 1 cells. Tritiated water release was linear with time up to 2 h. The $K_m$ was 70 nM and the $V_{max}$ was 7.1 pmol $^3H_2O$ released per mg of protein per hour, similar to the values of 55.6 nM and 10 pmol mg$^{-1}$ h$^{-1}$ reported by Zhou *et al*. (1990). Stably expressed aromatase activity was inhibited in Arom. 1 cells by both CGS 16949A and 4-hydroxyandrostenedione, with $IC_{50}$ values of 7.4 and 70 nM respectively. At 2 h, WT and Arom. 2 cells had no detectable aromatase activity. After 24 h incubation, aromatase activity was detected in WT (12 fmol mg$^{-1}$ protein 24 h$^{-1}$) and Arom. 2 (3 fmol mg$^{-1}$ 24 h$^{-1}$). This was approximately 1,000 times lower than that present in Arom. 1 (6120 fmol mg$^{-1}$ 24 h$^{-1}$).

*Morphology, ER expression, cell culture*

The light microscopic appearances of Arom. 1 and Arom. 2 were not significantly different from WT (not shown). ER expression was detected in WT, Arom. 1 and Arom. 2 cell lines at 130 fmol per mg of protein.

When cultured in complete medium (CM) there was no significant difference in the growth rates of WT, Arom. 1 or Arom. 2 cells (data not shown). However, a difference in growth rate was observed in steroid-depleted medium (SDM): after 6 days the number of Arom. 1 cells was significantly higher than the number of WT or Arom. 2 cells (Figure 2). Arom. 1 cells growing in SDM showed further growth enhancement in the presence of $10^{-12}$ to $10^{-7}$ M androstenedione or testosterone, with maximal stimulation (approximately 130–200% control after 6 days) at $10^{-11}$ to $10^{-10}$ M. This response to androgens was not manifest by WT or Arom. 2 cells. All three cell lines showed a similar mitogenic response to $E_2$ at $10^{-12}$ to $10^{-7}$ M, with maximal effects (200–500% control at 6 days) in the presence of $10^{-11}$ M $E_2$ (not shown).

In evaluating the growth characteristics of Arom. 1, we used the Arom. 2 clone as control, since this cell line, like Arom. 1, had undergone transfection and G418 selection, but did not express aromatase activity at levels greater than WT. Figure 3 shows the effect of androstenedione, testosterone or

**Figure 1** Southern, Northern and Western analysis of wild-type (WT) MCF-7 cells and G418-resistant clones Arom. 1 and Arom. 2. **a**, Approximately $10^7$ cultured cells were used to prepare genomic DNA by salting out (Miller *et al.*, 1988) using the Stratagene DNA preparation kit according to the manufacturer's instructions. Genomic DNA (10 μg) was digested with *Eco*RI (Boehringer Mannheim), electrophoresed on a 0.8% agarose gel and transferred to Hybond $N^+$ (Amersham). Bound DNA was hybridised with a $^{32}P$-labelled 2 kb *Eco*RI fragment of p3681. **b**, Total RNA was prepared from $10^7$ to $2 \times 10^7$ cells by the method of Chomczynski and Sacchi (1987). RNA (30 μg) was electrophoresed on a 1% agarose–formaldehyde gel, transferred to Hybond $N^+$ and probed with a $^{32}P$-labelled 1.5 kb aromatase insert made by digestion of p3681 with *Hind*III and *Not*I. **c**, For Western analysis, $2-3 \times 10^6$ cells were washed with phosphate-buffered saline, pelleted, freeze–thawed and sonicated on maximum power for 15 s. Samples were electrophoresed on a 10% SDS polyacrylamide gel, transferred to nitrocellulose membrane (Schleicher & Schuell) and probed with polyclonal antibody R-8-2 at 1:1000 dilution (Kitiwaki *et al.*, 1989). The second antibody was an anti-rabbit HRP conjugate (Promega), and detection was by enhanced chemiluminescence (Kricka, 1990) using ECL reagents (Amersham) according to the manufacturer's instructions. WT, wild-type MCF-7 cells; A1, Arom. 1 clone; A2 Arom. 2 clone. The size of molecular weight markers are shown to the left of each blot, in kb for **a** and **b** and kDa for **c**.

$E_2$ $10^{-11}$ M on the growth of Arom. 1 and Arom. 2 cells, in the presence or absence of 500 nM CGS 16949A. In Arom. 1 cells (Figure 3a), CGS 16949A suppressed basal growth ($100 \pm 2\%$ to $76 \pm 1\%$, $P < 0.01$) and inhibited the response to androstenedione ($125 \pm 1\%$ to $78 \pm 1\%$, $P < 0.01$) and testosterone ($186 \pm 4\%$ to $107 \pm 1\%$, $P < 0.01$). Arom. 2 cells showed a mitogenic response to $E_2$ ($160 \pm 4\%$, $P < 0.01$). As in Arom. 1 cells, this effect was reduced in the presence of CGS 16949A ($142 \pm 4\%$) although it remained significantly ($P < 0.01$) above control. However Arom. 2 cells showed no response to androstenedione or testosterone, and CGS 16949A had no inhibitory effect in basal or androgen-supplemented conditions (Figure 3b).

Culture in SDM with tritiated substrate permitted simultaneous assessment of growth and aromatase activity. The results of culturing Arom. 1 cells with [7-$^3$H]testosterone are shown in Figure 4. After 24 h culture in medium containing 1.6 nM [7-$^3$H]testosterone, product isolation analysis revealed that 46% of the substrate had been converted to $E_2$. Dihydrotestosterone, the product of 5α-reductase activity, has very similar mobility to $E_2$ on TLC, and in theory could contaminate the '$E_2$' peaks generated by Arom. 1. However, significant contamination is thought to be unlikely because little or no '$E_2$' was generated by Arom. 2 cells, which should have similar 5α-reductase activity (see below, Figure 4c). By day 6 Arom. 1 cells had converted 100% of the substrate to oestrogen (16% $E_1$, 84% $E_2$; Figure 4a). This was associated with significant enhancement of cell growth on day 6 ($4.6 \pm 0.2 \times 10^5$ cells per well compared with $1.6 \pm 0.1 \times 10^5$ in control cultures, $P<0.01$, Figure 4c). In both day 1 and day 6 samples, aromatisation of 1.6 nM [7-$^3$H]testosterone was completely inhibited by 500 nM CGS 16949A. Cell numbers were significantly lower than in the absence of CGS 16949A ($2.6 \pm 0.7$ *vs* $4.6 \pm 0.2 \times 10^5$, $P<0.01$), and not significantly different from the cell number in control cultures. The higher [7-$^3$H]testosterone concentration of 16 nM was almost completely converted to $E_2$ (82%) by day 1. On day 6, 63.5% of tritiated product was identified as $E_2$, and 22.5% was an unidentified peak. There was significant growth enhancement ($2.6 \pm 0.4 \times 10^5$ cells per well, $P<0.05$), although the effect here was less than in cultures supplemented with 1.6 nM [7-$^3$H]testosterone, suggesting production of a higher concentration of $E_2$ than the $10^{-11}$ to $10^{-9}$ M range previously found to be optimal. As at the lower substrate concentration, CGS 16949A completely suppressed conversion of testosterone to $E_2$ during the first 24 h. By day 6 there had been negligible conversion to $E_2$ (8%), 62.5% steroid remained as testosterone and 21.5% was in an unidentified peak. This suppression of aromatase activity was associated with growth delay ($1.1 \pm 0.20 \times 10^5$ *vs* $2.6 \pm 0.4 \times 10^5$ in the absence of CGS 16949A, $P<0.01$), with cell numbers falling to the level observed in control cultures.

Results of the parallel experiment on Arom. 2 are shown in Figure 4b and d. No significant aromatase activity was detected in these cells on day 1, or on day 6 at the lower [7-$^3$H]testosterone concentration, 1.6 nM. After 6 days in the presence of 16 nM substrate, there was 13% conversion to $E_2$. This was not suppressed by CGS 16949A. Arom. 2 cell numbers showed no significant differences from control in the presence or absence of [7-$^3$H]testosterone or aromatase inhibitor.

**Figure 2** Growth in steroid-depleted medium. WT, Arom. 1 or Arom. 2 cells were seeded in triplicate at $6 \times 10^4$ cells per well in SDM. After 6 days the cells were trypsinised and counted on a Coulter counter. Bars represent the mean ± s.e.m. of triplicate cell counts. *The number of Arom. 1 cells was significantly greater ($P<0.01$ by Dunnett's test) than WT or Arom. 2.

### Discussion

The aim of this study was to assess the biological significance of intratumoral aromatase expression, particularly in areas in which studies of clinical material have yielded conflicting data. To this end we first sought to confirm that transfected MCF-7 cells expressed aromatase, comparing the level of enzyme activity with that detectable in clinical samples of breast cancer.

Molecular analysis confirmed incorporation of aromatase cDNA into MCF-7 clones Arom. 1 and Arom. 2. In both clones the transfected plasmid was present at lower copy number than the endogenous aromatase gene. A similar finding, compatible with amplification of the endogenous gene, was made by Zhou *et al.* (1990) when expressing aromatase in mammalian cells for screening of new inhibitors. We detected high levels of expressed aromatase by Northern and Western analysis in clone Arom. 1 but not WT or Arom. 2. As we have previously reported (Ryde *et al.*, 1992), WT cells have detectable aromatase activity, which we and others (Zhou *et al.*, 1990; Pizzini *et al.*, 1992) found too low for full kinetic analysis. Despite incorporation of the expression construct, Arom. 2 cells did not have aromatase activity above WT levels. The reason for this is unclear.

Enzyme assay of Arom. 1 revealed a $K_m$ of 7.1 pmol mg$^{-1}$ h$^{-1}$. The results of 24 h assay showed these cells to express approximately 1,000 times more aromatase activity than WT or Arom. 2 cells. We observed 100% conversion of physiological levels of testosterone ($10^{-9}$ to $10^{-8}$ M) to oestrogens in Arom. 1 cells (Figure 4). This represents 100–1,000 times the level of aromatase activity in clinical tumour specimens (Varela & Dao, 1978; Miller *et al.*, 1982, 1990; Lipton

**Figure 3** Response of Arom. 1 or Arom. 2 cells to androgens, $E_2$ and CGS 16949A. Cells were grown for 6 days in SDM with no additives (control) or $10^{-11}$ M androstenedione, testosterone or $E_2$ in the absence or presence of 500 nM CGS 16949A. Bars represent the mean ± s.e.m. of triplicate counts. **a**, Arom. 1; **b**, Arom. 2.

**Figure 4** Aromatase activity and growth in the presence of [7-$^3$H]testosterone. **a** and **b** show product isolation analysis of culture supernatants from **a**, Arom. 1 and **b**, Arom. 2 after 1 and 6 days culture in SDM with 1.6 or 16 nM [7-$^3$H]testosterone in the absence or presence of 500 nM CGS 16949A. The bars represent the means of triplicate analyses; all s.e.m.s were <10% means. **c** and **d** show growth curves (mean ± s.e.m. of triplicate counts) for **c**, Arom. 1 and **d**, Arom. 2 in SDM with no additives (control) or 1.6 or 16 nM [7-$^3$H]testosterone alone or with 500 nM CGS 16949A. ⊡, control (no testosterone); ■, control + CGS; O, 1.6 nM testosterone; ●, 1.6 nM testosterone ± CGS; Δ, 16 nM testosterone; ▲, 16 nM testosterone + CGS.

*et al.*, 1987; Silva *et al.*, 1989). Enzyme activity in clinical tumour homogenates reflects aromatase expressed by the tumour cells, and at lower levels, by breast adipose cells (Perel *et al.*, 1982). Extracellular matrix makes no contribution; indeed aromatase activity is higher in highly cellular tumours than in those of moderate/low cellularity (Miller *et al.*, 1990). This hetergeneous expression pattern could be modelled *in vitro* or *in vivo* by mixing Arom. 1 with a 100- to 1,000-fold excess of WT or Arom. 2 cells.

Having characterised the aromatase activity of WT MCF-7 cells and the two transfected clones, we compared cells with high and low aromatase activity with regard to morphology, ER status, growth pattern and response to aromatase inhibitors. Although an excess of aromatase-positive tumours have been reported to be high grade (Silva *et al.*, 1989), Lipton *et al.* (1988) found no correlation between intratumoral aromatase expression and degree of differentiation. In support of this conclusion we found that overexpression of aromatase had no effect on the morphology of cultured cells. Similarly we found no difference in levels of ER expression between Arom. 1 and WT cells. Some clinical studies have found a positive correlation between intratumoral aromatase expression and ER positivity (Miller *et al.*, 1982, 1990; Bolufer *et al.*, 1992). Others suggest that aromatase-positive tumours are more often ER negative (Abul-Hajj *et al.*, 1979; Esteban *et al.*, 1992), and some have found no correlation (Varela & Dao, 1978; Lipton *et al.*, 1987; Bezwoda *et al.*, 1987*a*; Silva *et al.*, 1989).

Most breast cancers are thought to be hormone dependent early in their natural history (Lippman & Dickson, 1990), and so aromatase activity may affect the growth of the tumour. Local oestrogen production may enhance growth of the aromatase-positive cells and, in a paracrine fashion, adjacent aromatase-negative cells. Several studies have attempted to correlate aromatase activity with clinical prognostic factors and response to treatment (Bezwoda *et al.*, 1987*a*,*b*; Silva *et al.*, 1989; Miller *et al.*, 1990; Bolufer *et al.*, 1992). In our *in vitro* model we found no growth difference in complete medium, as serum provides sufficient oestrogen for optimal growth. However in SDM Arom. 1 cells grew faster than WT or Arom. 2 (Figure 2). We have no definite explanation for this observation, but it is consistent with a response to residual androgen remaining in SDM after charcoal stripping of serum. There was more marked growth enhancement on addition of androstenedione or testosterone. We did not observe a response to androgens in Arom. 2 or WT cells; neither cell line grew in the absence of exogenous oestrogen. However, there is evidence for heterogeneity of aromatase expression in WT MCF-7 cells, sufficient to permit some sublines to grow in androgen-supplemented SDM (Darbre & Daly, 1989; Kitiwaki *et al.*, 1992).

Finally we assessed the effect of aromatase inhibitors on WT, Arom. 1 and Arom. 2 cells. Both CGS 16949A and 4-hydroxyandrostenedione inhibited the aromatase activity of Arom. 1 cells, with $IC_{50}$ values (7.4 and 70 nM respectively) similar to results reported for human placental microsomes (5

and 62 nM; Bhatnagar et al., 1989). This suggests that the Arom. 1 cells may be suitable for use in the screening of new inhibitors. CGS 16949A suppressed basal growth of Arom. 1 cells to approximately 75% of control. This could have been due to direct toxicity, although there was no growth inhibition in Arom. 2 cells cultured under basal or androgen-supplemented conditions (Figure 3). Alternatively, it could be due to suppression of the mitogenic response to any residual androgen remaining in SDM, but we have no direct evidence for this. CGS 16949A also caused complete suppression of the mitogenic effect of $10^{-11}$ M androstenedione or testosterone. Experiments with tritiated substrates enable us to correlate directly aromatase activity and growth enhancement, and to assess the inhibition of enzyme activity and growth by CGS 16949A. Here we used androgen concentrations of 1.6–16 nM, equivalent to circulating levels in post-menopausal women (Judd et al., 1974). We did not purify 'oestrogens' separated by TLC, but significant contamination of these peaks with other products seemed unlikely for several reasons. The negligible loss of testosterone to other peaks (in particular those associated with $E_1$ or $E_2$) in Arom. 2 cells indicates little underlying metabolism of this precursor to oestrogens or other products coincident on TLC. In Arom. 1 cells there was good correlation between conversion of testosterone to $E_2$, which was generally complete after 24 h, and growth enhancement observed on day 6. CGS 16949A, a highly selective aromatase inhibitor, caused virtual obliteration of 'oestrogen' peaks generated by Arom. 1 cells. This was accompanied by complete suppression of androgen-stimulated growth, again supporting the view that oestrogens were the main products of testosterone metabolism by the Arom. 1 clone.

In summary we have developed a clone of MCF-7 cells which stably overexpresses aromatase at 1,000 times the level in WT cells. This had no detectable effect on morphology, ER expression or response to $E_2$. However, there was significant growth enhancement in steroid-depleted conditions, and this growth advantage over WT cells was further enhanced by exogenous androgens. These changes were fully reversible by aromatase inhibitors. We plan to evaluate the growth characteristics of Arom. 1 cells *in vivo*. The ability to manipulate aromatase expression by breast cancer xenografts should help to clarify the significance of intratumoral aromatase detectable in clinical breast tumours.

This work was supported by the Cancer Research Campaign and the Medical Research Council.

**Abbreviations:** $E_1$, oestrone; $E_2$, oestradiol; ER, oestrogen receptor; PCR, polymerase chain reaction; $K_m$, substrate concentration producing half-maximal velocity; $V_{max}$, maximal velocity; $IC_{50}$, drug concentration causing 50% inhibition of enzyme activity.

### References

ABUL-HAJJ, Y.J., IVERSON, R. & KIANG, D.T. (1979). Aromatization of androgens by human breast cancer. *Steroids*, **33**, 205–222.

ASHWORTH, A. (1993). Cloning transcription factors by homology. In *Transcription Factors: A Practical Approach*, Latchman, D.S. (ed.) pp. 125–142. Oxford University Press: Oxford.

BEZWODA, W.R., MANSOOR, N., DANSEY, R. & ESSER, J.D. (1987*a*). Aromatisation of androstenedione by human breast cancer tissue: correlation with hormone receptor activity and possible biologic significance. *Oncology*, **44**, 30–33.

BEZWODA, W.R., MANSOOR, N. & DANSEY, R. (1987*b*). Correlation of breast tumour aromatase activity and response to aromatase inhibition with aminoglutethimide. *Oncology*, **44**, 345–349.

BHATNAGAR, A.S., SCHIEWECK, K., HAUSLER, A., BROWN, L.J. & STEELE, R.E. (1989). Inhibitors of oestrogen biosynthesis: preclinical studies with CGS 16949A, a new nonsteroidal aromatase inhibitor. *Proc. R. Soc. Edin.*, **95B**, 293–303.

BOLUFER, P., RICART, E., LLUCH, A., VAZQUEZ, C., RODRIGUEZ, A., RUIZ, A., LLOPIS, F., GARCIA-CONDE, J. & ROMERO, R. (1992). Aromatase activity and estradiol in human breast cancer: its relationship to estradiol and epidermal growth factor receptors and to tumor-node-metastasis staging. *J. Clin. Oncol.*, **10**, 438–446.

BRADFORD, M.M. (1976). A rapid and sensitive method for the quantification of microgram quantities of protein using the principle of protein–dye binding. *Anal. Biochem*, **72**, 248–254.

BRADLOW, H.L. (1982). A reassessment of the role of breast tumor aromatization. *Cancer Res.*, **42**, (Suppl.) 3382s–3386s.

CHEN, T.R. (1977). *In situ* detection of mycoplasma contamination in cell cultures by fluorescent Hoechst 33258 stain. *Exp. Cell Res.*, **104**, 255–262.

CHOMCZYNSKI, P. & SACCHI, N. (1987). Single-step method of RNA isolation by acid guanidinium thiocyanate–phenol–chloroform extraction. *Anal. Biochem.*, **162**, 156–159.

CHU, G., HAYAKAWA, H. & BERG, P. (1987). Electroporation for the efficient transfection of mammalian cells with DNA. *Nucleic Acids Res.*, **15**, 1311–1326.

CORBIN, C.J., GRAHAM-LORENCE, S., MCPHAUL, M., MASON, J.I., MENDELSON, C.R. & SIMPSON, E.R. (1988). Isolation of a full-length cDNA insert encoding human aromatase system cytochrome P-450 and its expression in nonsteroidogenic cells. *Proc. Natl Acad. Sci. USA*, **85**, 8948–8952.

DARBRE, P.D. & DALY, R.J. (1989). Effects of oestrogen on human breast cancer cells in culture. *Proc. R. Soc. Edin.*, **95B**, 119–132.

ESTEBAN, J.M., WARSI, Z., HANIU, M., HALL, P., SHIVELY, J.E. & CHEN, S. (1992). Detection of intratumoral aromatase in breast carcinomas. An immunohistochemical study with clinicopathologic correlation. *Am. J. Pathol.*, **140**, 337–343.

FOURNET-DULGUEROV, N., MACLUSKY, N.J., LERANTH, C.Z., TODD, R., MENDELSON, C.R., SIMPSON, E.R. & NAFTOLIN, F. (1987). Immunohistochemical localization of aromatase cytochrome P-450 and estradiol dehydrogenase in the syncytiotrophoblast of the human placenta. *J. Clin. Endocrinol. Metab.*, **65**, 757–764.

JUDD, H.L., JUDD, G.E., LUCAS, W.E. & YEN, S.S.C. (1974). Endocrine function of the postmenopausal ovary: concentration of androgens and estrogens in ovarian and peripheral vein blood. *J. Clin. Endocrinol. Metab.*, **39**, 1020–1024.

KITIWAKI, J., YOSHIDA, N. & OSAWA, Y. (1989). An enzyme-linked immunosorbent assay for quantitation of aromatase cytochrome p-450. *Endocrinology*, **124**, 1417–1423.

KITIWAKI, J., FUKUOKA, M., YAMAMOTO, T., HONJO, H. & OKADA, H. (1992). Contribution of aromatase to the deoxyribonucleic acid synthesis of MCF-7 human breast cancer cells and its suppression by aromatase inhibitors. *J. Steroid Biochem. Mol. Biol.*, **42**, 267–277.

KRICK, L.J. (1990). Chemiluminescent and bioluminescent techniques. *Clin. Chem.*, **37**, 1472–1478.

LEPHART, E.D. & SIMPSON, E.R. (1991). Assay of aromatase activity. *Methods Enzymol.*, **206**, 477–483.

LIPPMAN, M.E. & DICKSON, R.B. (1990). Growth control of normal and malignant breast epithelium. *Prog. Clin. Biol. Res.*, **354A**, 147–178.

LIPTON, A., SANTNER, S.J., SANTEN, R.J., HARVEY, H.A., FEIL, P.D., WHITE-HERSHEY, D., BARTHOLOMEW, M.J. & ANTLE, C.E. (1987). Aromatase activity in primary and metastatic human breast cancer. *Cancer*, **59**, 779–782.

LIPTON, A., SANTEN, R.J., SANTNER, S.J., HARVEY, H.A., WHITE-HERSHEY, D., BARTHOLOMEW, M.J. & SHARKEY, F.E. (1988). Correlation of aromatase activity with histological differentiation of breast cancer – a morphometric analysis. *Breast Cancer Res. Treat.*, **12**, 31–35.

MACINDOE, J.H. (1979). Estradiol formation from testosterone by continuously cultured human breast cancer cells. *J. Clin. Endocrinol. Metab.*, **49**, 272–277.

MCNATTY, K.P., BAIRD, D.T., BOLTON, A., CHAMBERS, P., CORKER, C.S. & MCLEAN, H. (1976). Concentration of oestrogens and androgens in human ovarian venous plasma and follicular fluid throughout the menstrual cycle. *J. Endocrinol.*, **71**, 77–85.

MILLER, S.A., DYKES, D.D. & POLESKY, H.F. (1988). A simple salting out procedure for extracting DNA from human nucleated cells. *Nucleic Acids Res.*, **16**, 1215.

MILLER, W.R., HAWKINS, R.A. & FORREST, A.P.M. (1982). Significance of aromatase activity in human breast cancer. *Cancer Res.*, **42**, (Suppl.) 3365s–3368s.

MILLER, W.R., ANDERSON, T.J. & JACK, W.J.L. (1990). Relationship between tumour aromatase activity, tumour characteristics and response to therapy. *J. Steroid Biochem. Mol. Biol.*, **37**, 1055–1059.

PEREL, E., BLACKSTEIN, M.E. & KILLINGER, D.W. (1982). Aromatase in human breast carcinoma. *Cancer Res.*, **42**, (Suppl.) 3369s–3372s.

PIZZINI, A., BRIGNARDELLO, E., LEONARDI, L., DI MONACO, M. & BOCCUZZI, G. (1992). Aromatase fails to mediate the proliferative effects of adrenal androgens on cultured MCF-7 breast cancer cells. *Int. J. Oncol.*, **1**, 709–712.

RYDE, C.M., NICHOLLS, J.E. & DOWSETT, M. (1992). Steroid and growth factor modulation of aromatase activity in MCF-7 and T47D breast carcinoma cell lines. *Cancer Res.*, **52**, 1411–1415.

SEED, B. & ARUFFO, A. (1987). Molecular cloning of the CD2 antigen, the T-cell erythrocyte receptor, by a rapid immunoselection procedure. *Proc. Natl Acad. Sci. USA*, **84**, 3365–3369.

SILVA, M.C., ROWLANDS, M.G., DOWSETT, M., GUSTERSON, B., MCKINNA, J.A., FRYATT, I. & COOMBES, R.C. (1989). Intratumoral aromatase as a prognostic factor in human breast cancer. *Cancer Res.*, **49**, 2588–2591.

SIMPSON, E.R., MERRILL, J.C., HOLLUB, A.J., GRAHAM-LORENCE, S. & MENDELSON, C.R. (1989). Regulation of estrogen biosynthesis by human adipose cells. *Endocrinol. Rev.*, **10**, 136–148.

VARELA, R.M. & DAO, T.L. (1978). Estrogen synthesis and estradiol binding by human mammary tumors. *Cancer Res.*, **38**, 2429–2433.

ZHOU, D., POMPON, D. & CHEN, S. (1990). Stable expression of human aromatase complementary DNA in mammalian cells: a useful system for aromatase inhibitor screening. *Cancer Res.*, **50**, 6949–6954.